# TNFAIP8 interacts with LATS1 and promotes aggressiveness through regulation of Hippo pathway in hepatocellular carcinoma

**DOI:** 10.18632/oncotarget.14938

**Published:** 2017-02-01

**Authors:** Qianze Dong, Lin Fu, Yue Zhao, Chengyao Xie, Qingchang Li, Enhua Wang

**Affiliations:** ^1^ College of Basic Medical Sciences and Department of Pathology, First Affiliated Hospital, China Medical University Shenyang, Liaoning, China

**Keywords:** TNFAIP8, hepatocellular carcinoma, YAP, Hippo

## Abstract

Although TNFAIP8 overexpression has been implicated in several human cancers, its clinical significance and biological function in hepatocellular carcinoma (HCC) remains unknown. Our study demonstrated that TNFAIP8 overexpression in primary HCC samples correlated with TNM stage, recurrence, poor prognosis and served as an independent favorable prognostic factor. We further showed that TNFAIP8 upregulated cell proliferation, migration, invasion and xenograft tumor growth of HCC cells. In addition, TNFAIP8 overexpression inhibited YAP phosphorylation, increased its nuclear localization and stabilization, leading to upregulation of cyclin proteins, CTGF and cell proliferation. We also found that TNFAIP8 could interact with LATS1 and decreased its phosphorylation. Depletion of LATS1 and YAP by siRNA blocked the biological effects of TNFAIP8. Collectively, the present study provides a novel finding that TNFAIP8 promotes HCC progression through LATS1-YAP signaling pathway. TNFAIP8 may serve as a candidate biomarker for poor prognosis and a target for new therapies.

## INTRODUCTION

Hepatocellular carcinoma (HCC) is the third most common cause of cancer mortality worldwide and the incidence of this cancer is increasing [1]. Despite recent advances in the treatment, the survival of HCC patients remains poor [2–4]. Many molecular markers that affect aggressiveness of HCC [5–8]. It is important to identify new markers and their molecular mechanisms involved in the regulation of HCC aggressiveness.

The Hippo signaling pathway promotes contact inhibition of cell growth and inducces of apoptosis [9, 10]. Dysregulation of Hippo signaling pathway has been indicated in hepatocellular carcinoma [11, 12]. Loss of Hippo signaling leads to YAP nuclear accumulation and eventually liver tumor in a transgenic mouse model [13]. Hippo effector YAP is overexpressed in various cancers and functions as an oncogene [14–18].

TNFAIP8, also known as SCC-S2, has been identified in human head and neck squamous cell carcinoma (HNSCC) [19]. TNFAIP8 could be induced by TNF-α and activation of NF-κB signaling pathways [20, 21]. TNFAIP8 promoted cell survival by downregulation of caspase-3 and caspase-8 activity [22]. TNFAIP8 is also overexpressed in cervical, ovarian, prostate, gastric carcinoma and promotes cancer cell growth and invasion [23–28]. These data suggests TNFAIP8 as an oncoprotein in human cancer development. However, the molecular mechanism of its biological function remains controversial.

In this study, we demonstrated that TNFAIP8 protein was upregulated in human hepatocellular carcinoma and associated with malignant clinical parameters. We also investigated its biological effects and the potential molecular mechanism in HCC cell lines.

## RESULTS

### Expression of TNFAIP8 correlates with clinicopathological factors and poor prognosis in HCC patients

**Figure 1:**
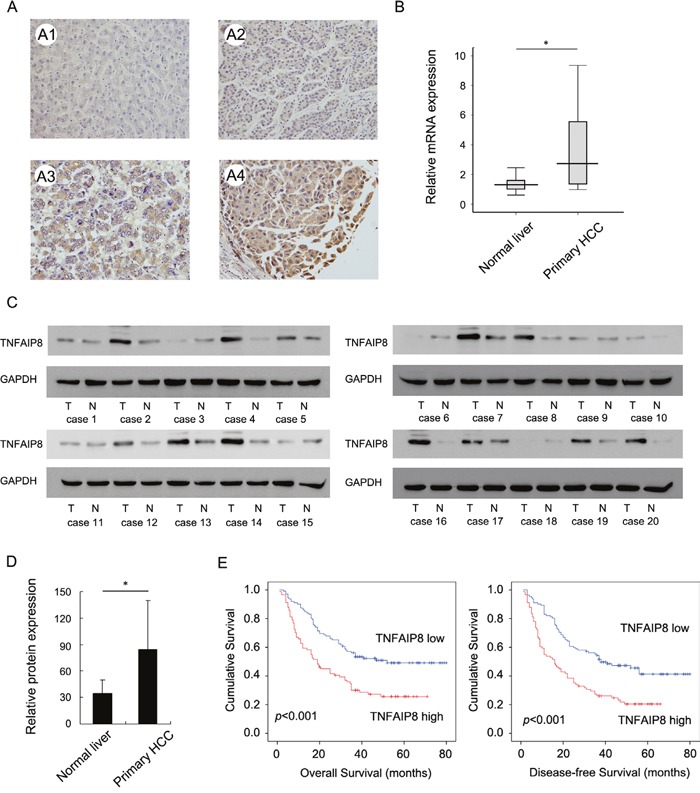
Clinical significance of TNFAIP8 in hepatocellular carcinoma **A**. Expression of TNFAIP8 protein in normal liver tissue and hepatocellular carcinoma. A1. Negative TNFAIP8 in normal liver tissues. A2. Weak TNFAIP8 staining in HCC tissue. A3. Moderate cytoplasmic TNFAIP8 staining in HCC tissue. A4. Strong cytoplasmic TNFAIP8 staining in HCC tissue. **B**. Real-time PCR showed that mean TNFAIP8 mRNA level in 20 fresh HCC tissues was higher than that in paired adjacent normal tissues. **C**. Western blot analysis showed TNFAIP8 protein was significantly elevated in 12/20 of HCC tissues compared with corresponding normal tissues. **D**. Mean value of TNFAIP8 intensity in 20 cases of fresh HCC tissues was higher than that in adjacent normal tissues. **E**. Kaplan-Meier analysis showed patients with high TNFAIP8 had worse overall survival and disease-free survival than patients with low TNFAIP8 protein.

To explore clinical and prognostic significance of TNFAIP8 in HCC patients, we examined its protein expression in 203 HCC specimens using immunohistochemistry. Immunohistochemistry data showed that TNFAIP8 was primarily localized to the cytoplasm. TNFAIP8 expression was significantly elevated in 91 out of 203 (41.1%) primary HCC samples. Normal tissues showed weak/negative TNFAIP8 staining (Figure 1A). We also examined its protein and mRNA levels in 20 fresh HCC tissues and paired adjacent normal tissues. Real-time PCR analysis showed that TNFAIP8 mRNA level was much higher in HCC tissues compared with that in adjacent normal tissues (Figure 1B & Supplementary Figure S1). Western blot analysis showed significant TNFAIP8 protein overexpression in 12/20 of HCC tissues (Figure 1C & Supplementary Figure S1). We analyzed relative grey value of western blot bands and the mean value of TNFAIP8 intensity in HCC tissues was significant higher than that in adjacent normal tissues (Figure 1D).

To explore the clinical significance of TNFAIP8 in HCC tissues, we analyzed the relationship between TNFAIP8 expression and clinicopathological parameters. As summarized in Table 1, there was no significant associations between TNFAIP8 overexpression and age, gender, tumor number, cirrhosis, serum AFP, HBV or differentiation. Tumors with TNFAIP8 overexpression tended to display more aggressive phenotypes, including larger tumor size (p=0.0056), advanced TNM stage (p=0.0180) and higher recurrence rate (p=0.0038).

**Table 1:** Distribution of TNFAIP8 status in hepatocellular carcinoma according to clinicopathological characteristics

Characteristics	Number of patients	TNFAIP8 low expression	TNFAIP8 high expression	*P*
Gender				
Female	31	19	12	0.4568
Male	172	93	79	
Age				
≤60	150	86	64	0.2976
>60	53	26	27	
Tumor number				
Single	171	93	78	0.6025
Multiple	32	19	13	
Tumor Size				
≤5 cm	77	52	25	0.0056
>5 cm	126	60	66	
Cirrhosis				
Absent	129	72	57	0.8083
Present	74	40	34	
AFP (ng/ml)				
<20	53	28	25	0.6900
≥20	150	84	66	
HBV				
Negative	43	25	18	0.6594
Positive	160	87	73	
Tumor differentiation				
I+II	132	75	57	0.5203
III+IV	71	37	34	
TNM stage				
I+II	99	63	36	0.0180
III	104	49	55	
Recurrence				
No	127	80	47	0.0038
Yes	76	32	44	

The Kaplan-Meier analysis showed that patients with high TNFAIP8 expression had shorter overall survival and disease-free survival than those with low TNFAIP8 expression (Figure 1E). TNFAIP8 also correlated with poor overall survival in HCC subgroups with single tumor and tumor size >5cm (Supplementary Figure S2).

Univariate analysis revealed that tumor size, recurrence, TNM stage and TNFAIP8 expression were statistically correlated with patients' survival. These parameters were further subjected to a multivariate Cox proportional hazards model, which indicated that TNM stage, recurrence and TNFAIP8 were independent and significant factors for prognosis (Table 2).

**Table 2:** Univariate and Multivariate analysis for predictive factors in patients with hepatocellular carcinoma (Cox regression model)

Factors	Univariate		Multivariate	
	Hazard ratio(95% CI)	p value	Hazard ratio(95% CI)	p value
TNFAIP8	2.077 (1.438-2.999)	<0.001	1.671 (1.149-2.430)	0.0072
TNM stage	2.992 (2.020-4.431)	<0.001	1.839 (1.272-2.658)	0.0012
Cirrhosis	0.850 (0.578-1.250)	0.4098	1.130 (0.750-1.702)	0.5582
Tumor grade	1.184 (0.812-1.727)	0.3788	0.893 (0.596-1.338)	0.5825
Tumor size	2.249 (1.481-3.414)	<0.001	1.252 (0.795-1.974)	0.3322
Recurrence	2.647 (1.829-3.830)	<0.001	1.775 (1.197-2.633)	0.0043

### TNFAIP8 promotes proliferation, invasion and migration in HCC cell lines

**Figure 2:**
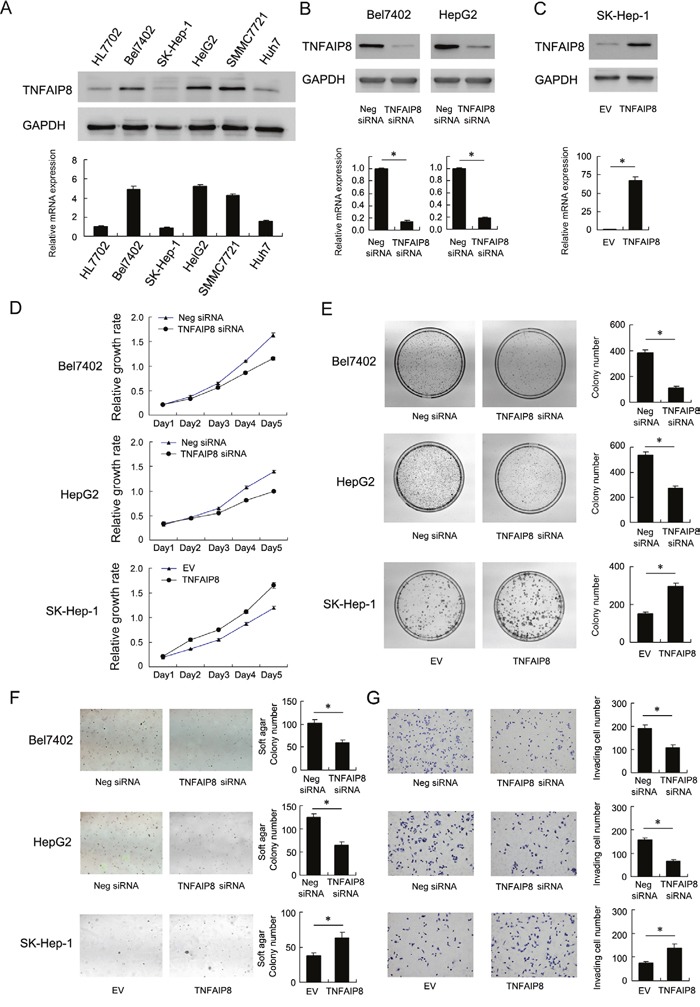
Biological roles of TNFAIP8 in hepatocellular carcinoma cell lines **A**. mRNA and protein levels of TNFAIP8 in a panel of HCC cell lines (including Bel7402, SK-Hep-1, HepG2, SMMC7721, Huh7) and normal cell line HL7702. **B**. siRNA transfection downregulated TNFAIP8 mRNA and protein in both Bel7402 and HepG2 cell lines. **C**. Plasmid transfection upregulated TNFAIP8 mRNA and protein in SK-Hep-1 cells. **D**. MTT showed time-dependent decrease in cell proliferation after TNFAIP8 siRNA transfection. TNFAIP8 plasmid transfection upregulated cell proliferation rate. **E**. TNFAIP8 siRNA decreased colony formation ability in Bel7402 (control vs TNFAIP8 siRNA: 380.6±22.8 vs 114.3±6.8, p<0.05) and HepG2 (control vs TNFAIP8 siRNA: 537.3±24.5 vs 273.3±19.3, p<0.05) cells. TNFAIP8 plasmid increased colony formation ability in SK-Hep-1 (EV vs TNFAIP8 plasmid: 150.3±9.8 vs 295±17.1, p<0.05) cells. **F**. Soft agar colony formation showed that TNFAIP8 depletion downregulated anchorage-independent cell growth in Bel7402 (control vs TNFAIP8 siRNA: 102±7.9 vs 59.3±6.5, p<0.05) and HepG2 (control vs TNFAIP8 siRNA: 125.3±7.6 vs 64.6±7.7, p<0.05) cell lines. TNFAIP8 overexpression upregulated anchorage-independent cell growth in SK-Hep-1 cells (EV vs TNFAIP8 plasmid: 37.6±4.5 vs 62.6±8, p<0.05). **G**. Matrigel invasion assay showed that TNFAIP8 siRNA downregulated cell invasion (Bel7402 control vs TNFAIP8 siRNA: 192±013.8 vs 109.3±10.4, p<0.05; HepG2 control vs TNFAIP8 siRNA: 157.6±8.3 vs 66±8.1, p<0.05), while its overexpression upregulated invading ability of SK-Hep-1 cells (EV vs TNFAIP8 plasmid: 75.6±6.1 vs 139.3±15.9, p<0.05).

To determine its biological role in HCC cells, plasmid and siRNA transfection were performed. We first analyzed its mRNA and protein levels in a panel of HCC cell lines and normal cell line HL7702. As shown in Figure 2A, TNFAIP8 expression in HCC cell lines was significantly higher compared with HL7702. Thus we employed siRNA to knockdown TNFAIP8 in Bel7402 and HepG2 cell lines. TNFAIP8 plasmid transfection was performed in SK-Hep-1 cell line. siRNA considerably reduced mRNA and protein expression after 48 hours of transfection (Figure 2B). TNAFIP8 plasmid significantly upregulated its mRNA and protein (Figure 2C).

MTT assay showed that TNFAIP8 depletion slowed down while its overexpression facilitated HCC cell growth rate *in vitro* (Figure 2D). Depletion of TNFAIP8 led to a significant reduction in colony numbers while overexpression upregulated colony number (Figure 2E). In addition, TNFAIP8 depletion inhibited anchorage independent cell growth in Bel7402 and HepG2 cell lines while its overexpression promoted soft agar colony formation in SK-Hep-1 cells (Figure 2F). We further examined the change of invasion and migration. As shown in Figure 2G, TNFAIP8 depletion inhibited invasion while its overexpression promoted cell invasion in HCC cells. Wound healing assay showed that depletion of TNFAIP8 attenuated cell migration while its overexpression facilitated cell migration (Supplementary Figure S3).

### TNFAIP8 regulates cell cycle progression and CTGF in HCC cell lines

**Figure 3:**
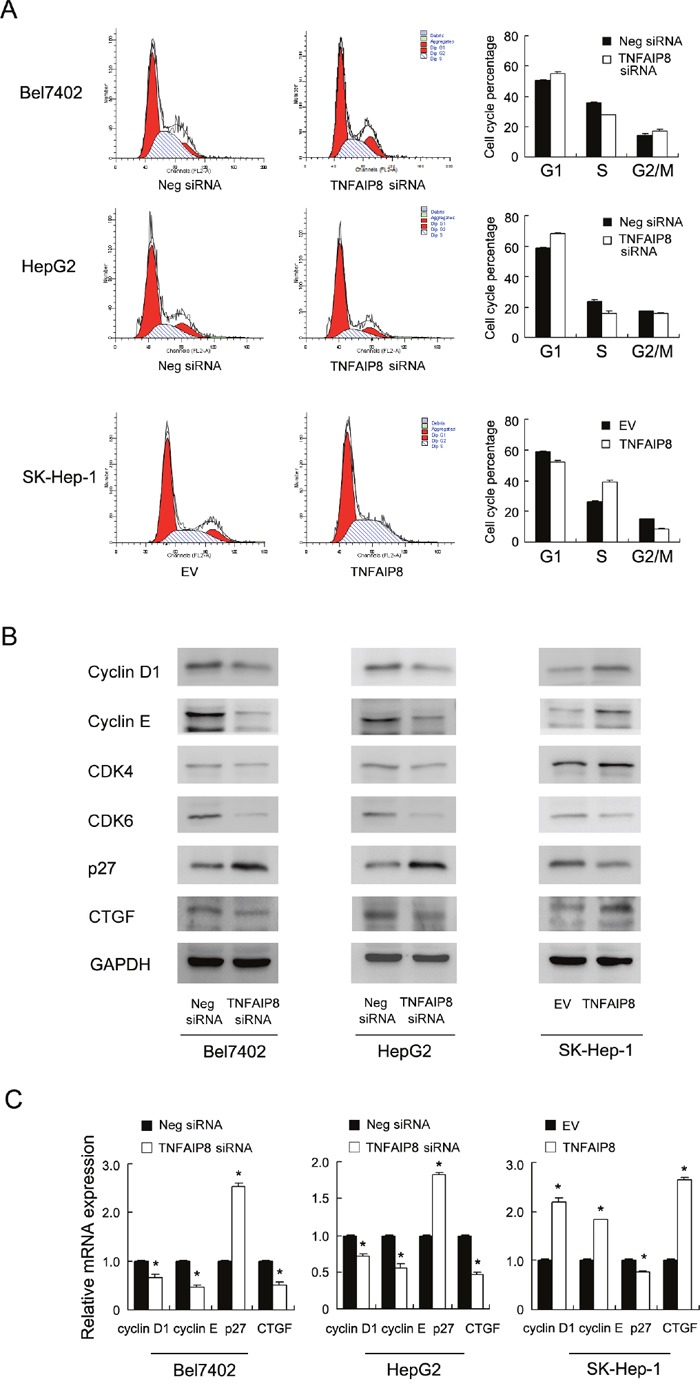
TNFAIP8 regulates cell cycle progression and related proteins **A**. Flow cytometry showed that TNFAIP8 depletion downregulated S phase percentage (Bel7402, control vs TNFAIP8 siRNA, 35.5%±0.6 vs 27.5%±0.4, p<0.05; HepG2, control vs TNFAIP8 siRNA, 23.7%±0.8 vs 16.1%±1.2, p<0.05) and upregulated G1 phase percentage (Bel7402, control vs TNFAIP8 siRNA, 50.5%±0.5 vs 55.3%±1.1, p<0.05; HepG2, control vs TNFAIP8 siRNA, 58.6%±0.6 vs 68.3%±0.7, p<0.05). TNFAIP8 overexpression upregulated S phase percentage (SK-Hep-1, control vs TNFAIP8 plasmid, 26.1%±0.7 vs 39.1%±0.7, p<0.05) and downregulated G1 phase (SK-Hep-1, control vs TNFAIP8 plasmid, 58.9%±0.4 vs 52.2%±0.6, p<0.05). **B**. western blot revealed that knockdown of TNFAIP8 depletion downregulated the protein levels of cyclin D1, cyclin E, CTGF while upregulated p27 in both Bel7402 and HepG2 cell lines. In TNFAIP8 transfected SK-Hep-1 cells, the levels of cyclin D1, cyclin E and CTGF were increased, with downregulation of p27 protein. **C**. Realtime PCR showed that TNFAIP8 siRNA downregulated cyclin D1, cyclin E and CTGF mRNA while upregulated p27 mRNA. TNFAIP8 overexpression showed the opposite effects in SK-Hep-1 cell line.

Cell cycle analysis was performed and the results showed that TNFAIP8 knockdown increased G1 phase percentage while decreased S phase percentage. TNFAIP8 overexpression increased S phase percentage while decreased G1 phase cells. These results demonstrate that TNFAIP8 positively regulates cell cycle progression at the G1/S boundary. To investigate the mechanism we examined change of related proteins including cyclin D1, cyclin E, p27, CDK4, CDK6. As shown in Figure 3B, western blot revealed that knockdown of TNFAIP8 downregulated cyclin D1, cyclin E while upregulated p27. In TNFAIP8 transfected SK-Hep-1 cells, the levels of cyclin D1 and cyclin E were increased, with downregulation of p27 protein. RT-qPCR analysis of these cell cycle regulator showed similar results (Figure 3C). Taken together, these results suggest that TNFAIP8 induces cell cycle progression during G1-S transition. In addition, we found that CTGF, a growth and invasion related protein, was significant downregulated after TNFAIP8 depletion and upregulated after TNFAIP8 overexpression (Figure 3B & 3C).

### TNFAIP8 depletion is associated with increased YAP phosphorylation and decreased YAP nuclear localization in HCC cell lines

**Figure 4:**
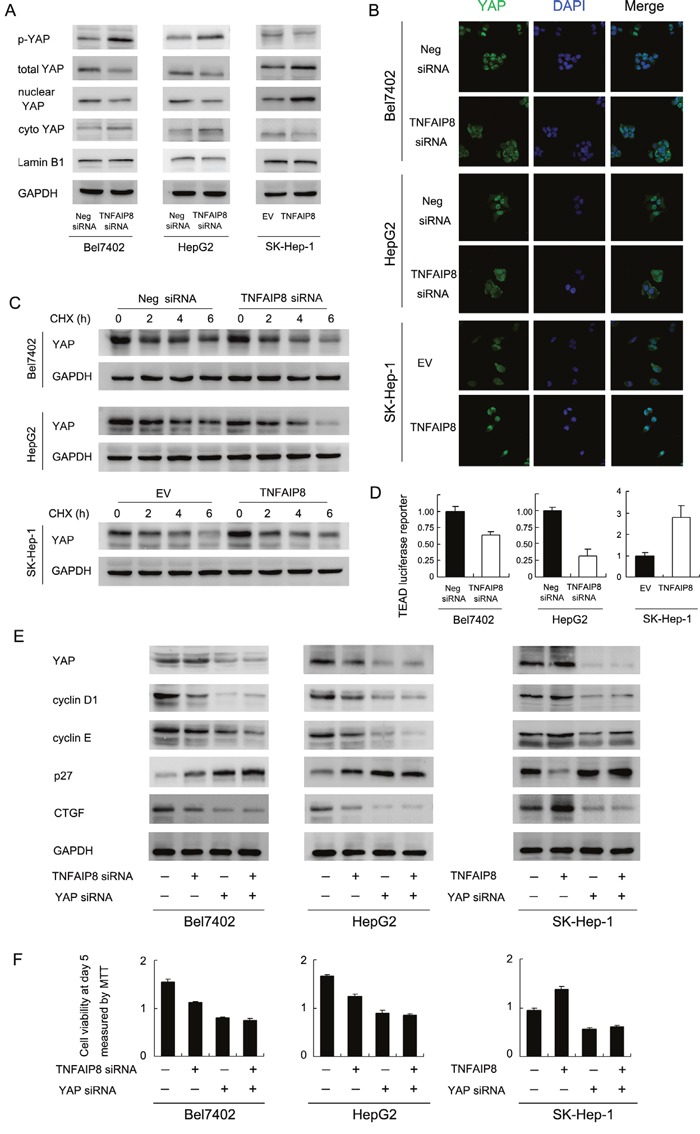
TNFAIP8 regulates YAP and Hippo signaling pathway **A**. TNFAIP8 siRNA treatment upregulated phospho-YAP while downregulated total and nuclear YAP protein. TNFAIP8 overexpression downregulated phospho-YAP while upregulated totol and nuclear YAP protein. **B**. Immunofluorescence showed that TNFAIP8 increased YAP nuclear localization while TNFAIP8 siRNA downregulated YAP nuclear localization. **C**. Using cycloheximide treatment, the stability of YAP protein was higher in TNFAIP8 transfected cells. TNFAIP8 depletion downregulated YAP stability. **D**. Using TEAD 8xGTIIC-luciferase reporter, TNFAIP8 upregulated transcriptional activity of YAP/TEAD in SK-Hep-1 cells. TNFAIP8 siRNA downregulated YAP/TEAD activity in both Bel7402 and HepG2 cell lines. **E**. YAP siRNA significantly downregulated YAP protein, cyclin D1, cyclin E, CTGF and upregulated p27. YAP siRNA treatment blocked the effect of TNFAIP8 overexpression or depletion on CTGF and cell cycle proteins. **F**. YAP siRNA blocked the effect of TNFAIP8 overexpression or depletion on cell growth rate.

CTGF is a downstream target of Hippo signaling pathway. Thus we checked the change of YAP protein, which serves as a Hippo effector. As shown in Figure 4A, TNFAIP8 overexpression increased total YAP protein and decreased YAP phosphorylation (Figure 4A). Immunofluorescence and western blot using nuclear/cytoplasmic fractionation showed nuclear YAP was downregulated after siRNA treatment while plasmid transfection upregulated YAP nuclear localization (Figure 4A and 4B).

Nuclear/cytoplasmic distribution and phosphorylation of YAP significantly influence its stabilization. To find out if TNFAIP8 could stabilize YAP protein, we treated HCC cells with protein synthesis inhibitor cycloheximide (CHX) after 40 hours of transfection. Relative protein intensity was shown in Supplementary Figure S4A. We observed that, after 6 hours of CHX treatment, TNFAIP8 overexpression significantly upregulated total YAP protein. TNFAIP8 siRNA showed the opposite effects (Figure 4C & Supplementary Figure S4A). These results indicate that the half-life of endogenous YAP increased with TNFAIP8 transfection and decreased after siRNA treatment. Then we performed luciferase reporter assay using reporter plasmid with 8xGTIIC-luciferase. The activity of TEAD luciferase reporter indicates transcriptional activity of YAP. Upregulation of TEAD luciferase activity correlates with inhibition of Hippo signaling pathway. As shown in Figure 4D, TNFAIP8 positively regulated transcription of YAP/TEAD. Based on the above results, we assumed that TNFAIP8 could stabilize the YAP protein and thus increase its steady-state protein level and nuclear localization, leading to inhibition of Hippo signaling.

Next, we asked whether YAP mediates TNFAIP8 induction of CTGF and cell cycle proteins. We adopted YAP siRNA to deplete its endogenous expression and test the effects of TNFAIP8. As shown in Figure 4E, siRNA significantly downregulated YAP protein, cyclin D1, cyclin E and CTGF and upregulated p27. In YAP depleted cells, the change of CTGF and cell cycle proteins was not significant (Figure 4E). YAP siRNA also reduced the effect of TNFAIP8 siRNA/plasmid on HCC cell proliferation (Figure 4F). In addition, YAP depletion block the role of TNFAIP8 on YAP/TEAD luciferase activity (Supplementary Figure S4B). Together, these results suggest that TNFAIP8 regulates HCC cell growth and invasion through regulation of Hippo effector YAP.

### TNFAIP8 regulates Hippo signaling through its interaction with LATS1

**Figure 5:**
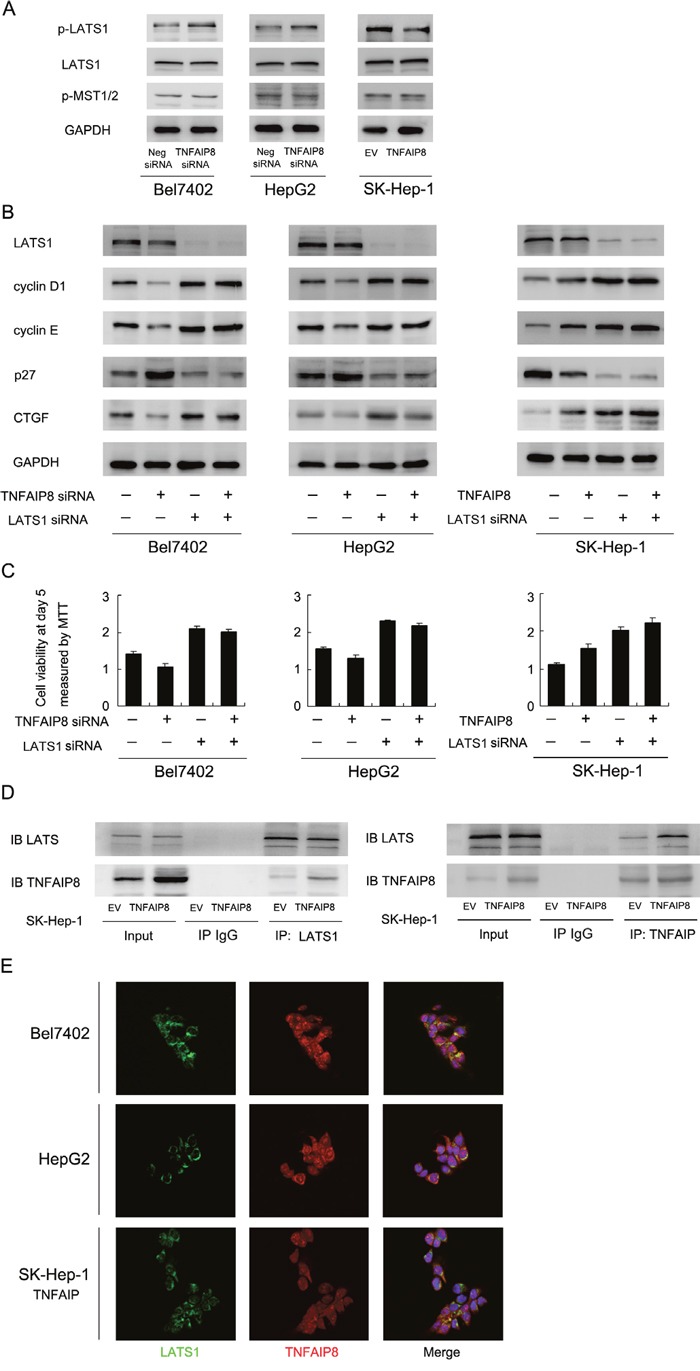
TNFAIP8 regulates Hippo signaling through interaction with LATS1 **A**. Depletion of TNFAIP8 upregulated LATS1 phosphorylation in Bel7402 and HepG2 cells. Its overexpression downregulated LATS1 phosphorylation in SK-Hep-1 cells. Change of MST1/2 phosphorylation was not significant. **B**. LATS1 siRNA significantly downregulated LATS1 protein, p27 and upregulated cyclin D1, cyclin E and CTGF. LATS1 siRNA treatment significantly reduced the effect of TNFAIP8 overexpression or depletion on CTGF and cell cycle proteins. **C**. LATS1 siRNA significantly reduced the effect of TNFAIP8 overexpression or depletion on cell growth rate. **D**. Co-immunoprecipitation analysis was performed in SK-Hep-1 cell lines. Note that LATS1 could co-immunoprecipitated with TNFAIP8. We also confirmed the association using a reciprocal approach. **E**. Immunofluorescence showed that TNFAIP8 could co-localize with LATS1 in HCC cell lines.

We further explored the possible mechanism of YAP change induced by TNFAIP8. YAP acts downstream of LATS1 and MST1/2 in Hippo signaling. Thus we checked status of these Hippo components. Western blot showed that TNFAIP8 depletion enhanced LATS1 phosphorylation, without significant change of p-MST1/2 (Figure 5A). Then we asked whether LATS1 mediates the roles of TNFAIP8 on YAP and its downstream proteins. LATS1 siRNA was employed to deplete endogenous LATS1. As shown in Figure 5B, in LATS1 siRNA treated cells, the effects of TNFAIP8 on CTGF and cell cycle proteins was obviously reduced. LATS1 siRNA treatment also significantly reduced the effects of TNFAIP8 siRNA on these proteins (Figure 5B). In addition, LATS1 significantly reduced the effect of TNFAIP8 on cell proliferation (Figure 5C). Together, these results suggest that the effect of TNFAIP8 on YAP and cell growth was dependent on, at least partly, regulation of LATS1 phosphorylation. Furthermore, co-immunoprecipitation was also carried out to examine if there is any interaction between TNFAIP8 and LATS1. As shown in Figure 5D, TNFAIP8 and LATS1 co-immunoprecipitated in these SK-Hep-1 cells with TNFAIP8 overexpression. To check if there is co-localization of these two proteins, we performed immunofluorescence in HCC cell lines. As shown in Figure 5E, co-localization of TNFAIP8 and LATS1 proteins was observed in Bel7402, HepG2 and SK-Hep-1/TNFAIP8 cells.

### TNFAIP8 promotes HCC cell growth *in vivo* and correlates with YAP/p-LATS1 in HCC tissues

**Figure 6:**
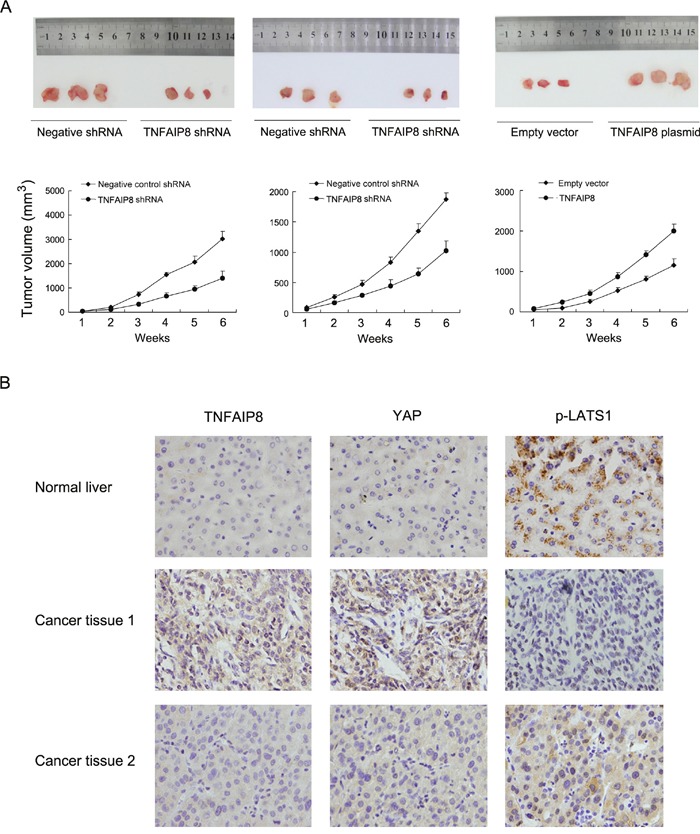
TNFAIP8 promotes HCC cell growth *in vivo* **A**. Nude mice bearing subcutaneous HCC cell xenografts were sacrifice after 6 weeks, Tissues were completely removed, and photos were taken. Tumor growth and volume of xenografts were significantly inhibited when injected with TNFAIP8 depleted cells compared with control. **B**. TNFAIP8 and YAP staining were negative in normal liver tissues while p-LATS1 showed positive cytoplasmic staining. In HCC tissues, cases with high TNFAIP8 protein expression tends to show strong YAP nuclear staining and low p-LATS1 expression.

To investigate the effects of TNFAIP8 *in vivo*, we established stable TNFAIP8 shRNA transfected Bel7402 and HepG2 cell lines and stable TNFAIP8 overexpressed SK-Hep-1 cell line. These cells were delivered by subcutaneous injection into the flanks of mice. As shown in Figure 6A, the rates of tumor growth of TNFAIP8 depleted Bel7402 and HepG2 were decreased compared with control. On the other hand, TNFAIP8-transfected SK-Hep-1 cells showed increased growth speed compared with empty vector.

To further validate the association between TNFAIP8 and Hippo signaling in HCC tissues. We examined the relationship between TNFAIP8 protein expression and Hippo components YAP and p-LATS in HCC specimens using immunohistochemistry. As shown in Figure 6B, both TNFAIP8 and YAP staining was negative in normal liver tissues while p-LATS1 showed positive cytoplasmic staining. In cancer tissues, cases with high TNFAIP8 protein expression tended to show strong YAP nuclear staining and low p-LATS1 (Table 3).

**Table 3:** Correlation of TNFAIP8 with YAP and p-LATS1 in HCC tissues

Characteristics	Number of patients	TNFAIP8 low expression	TNFAIP8 high expression	*P*
YAP				
Low expression	72	57	15	<0.001
High expression	131	55	76	
p-LATS1				
Low expression	150	97	53	<0.001
High expression	53	15	38	

## DISCUSSION

The involvement of Hippo dysregulation in human cancer development was originally verified in a conditional YAP transgenic mouse model, which develops liver nodules and eventually liver tumors [13]. Although many achievements have been made in elucidating Hippo signaling pathway, little is known about proteins that act upstream of Hippo signaling. In this study, we find out a new Hippo regulator, which interacts with LATS1 and inhibits its phosphorylation, leading to nuclear accumulation of YAP.

Overexpression of TNFAIP8 has been reported in several cancers [24–27, 29, 30]. Using immunohistochemistry, western blot and realtime PCR, we showed that TNFAIP8 was significantly upregulated in human HCC tissues, which correlated with larger tumor size, advanced TNM stage and recurrence, decreased overall and disease-free survival. Its prognostic value is also significant in sub-cohorts of patients with >5cm tumor and patients with single tumor. Furthermore, a multivariate analysis revealed that TNFAIP8 was an independent risk factor for poor survival of human HCC patients after curative resection. Our clinical data is in accordance with previous reports [25, 26, 28], demonstrating TNFAIP8 contributes to the malignant progression and its signature might have clinical values in predicting and providing prognostic information.

Our biological results demonstrated TNFAIP8 promoted HCC cell growth *in vitro* and *in vivo*, which was in line with previous studies [24–26]. We observed that TNFAIP8 facilitated cell cycle transition and regulated related proteins (including cyclin D1, cyclin E, CDK4/6, p27), suggesting TNFAIP8 control malignant cell growth through cell cycle regulation. Surprisingly, we found TNFAIP8 could induce CTGF mRNA and protein. CTGF (Connective tissue growth factor), which ranks among top upregulated genes by Hippo transducer YAP, which could lead to the activation of cyclin proteins and downregulation of p27 [31, 32]. CTGF positively regulates cancer invasion and migration through modulation of MMPs [33] and integrin signaling [34]. We believed that TNFAIP8 promotes aggressive behavior of HCC cell through, at least partly, regulation of YAP target protein CTGF.

Next, we sought to investigate whether and how TNFAIP8 modulate YAP and Hippo status. YAP is overexpressed at a high frequency in various human cancers [35–37]. We found that TNFAIP8 upregulated while its depletion downregulated total and nuclear YAP protein. TNFAIP8 also upregulated on TEAD luciferase reporter activity, which serves as a marker of YAP downstream function. It is well established that YAP status and function is controlled by phosphorylation. Phosphorylated YAP then binds to 14-3-3 protein and remains in the cytoplasm for degradation [38, 39]. Dephosphorylated YAP translocates into the nucleus and binds to TEAD proteins, which activates downstream target such as CTGF and promotes proliferation [38, 39]. Accordingly, TNFAIP8 reduced YAP phosphorylation. CHX treatment demonstrated TNFAIP8 overexpression inhibited YAP protein degradation. To validate the essential role of YAP in TNFAIP8 induced effects, we adopted YAP siRNA. The effects of TNFAIP8 on HCC cell growth, TEAD reporter activity and related proteins were diminished in YAP depleted cells. Taken together, these observations demonstrate that TNFAIP8 regulate HCC aggressiveness mainly through YAP and Hippo signaling pathway.

YAP phosphorylation is regulated by its interactions with other proteins including LATS1, MST1/2 and AMOT [40, 41]. Our results showed TNFAIP8 overexpression downregulated LATS1 phosphorylation, without significant change of MST1/2 phosphorylation. LATS1 depletion significantly reduced the effects of TNFAIP8 on cell cycle proteins and CTGF, as well as cell growth rate. Furthermore, we showed that TNFAIP8 could interact and co-localized with LATS1 in HCC cells. Our results support a model that LATS1 is responsible for TNFAIP8 induced change of cancer cell growth and Hippo signaling, which is further supported by the fact of TNFAIP8/LATS1 interaction. The correlation of TNFAIP8 with LATS1 and YAP in clinical samples further supports this hypothesis.

To conclude, our study supports that TNFAIP8 plays a major role during HCC progression. TNFAIP8 exerts its oncogenic function by interacting with LATS1, inhibiting its phosphorylation, restricting YAP in the nucleus and increasing its downstream targets, which promotes malignant phenotype of HCC. TNFAIP8 might serve as a candidate biomarker for prognosis and a target for new therapies.

## MATERIALS AND METHODS

### Patients and specimens

Primary tumor specimens were obtained from 203 patients diagnosed with hepatocellular carcinoma who underwent complete resection in the pathology archive of First Affiliated Hospital and Shengjing hospital of China Medical University between 2008 and 2012. Follow-up information was obtained by reviewing patients’ medical records. Patients without follow-up information were excluded. Informed consent was obtained from each patient, and the study protocol was approved by the Ethics Committee of China Medical University Institutional Board.

### Cell culture and transfection

HL-7702, Bel-7402, SK-Hep-1, HepG2, Huh7 and SMMC7721 cell lines were from American Type Culture Collection (Manassas, VA, USA). Cells were cultured in RPMI 1640 (Invitrogen, Carlsbad, CA, USA) containing 10% fetal calf serum.

siGENOME SMARTpool siRNA for TNFAIP8 (M-020589-01-0010), YAP (M-012200-00-0005), LATS1 (M-004632-00-0005) which contain a mixture of four siRNAs and siGENOME Non-targeting siRNA pool#1 (D-001206-13-20) were purchased from Dharmacon (Lafayette, CO, USA). The cells were transfected with siRNA using the DharmaFECT 1 transfection reagent according to the manufacturer's protocol.

On the basis of the TNFAIP8 sequence, short hairpin (sh)RNA was designed using the rnaidesigner (Invitrogen, USA): shTNFAIP8, 5′-GCCCAGATTGCATACTCTAAG-3′. Vectors encoding shRNA was generated using pENTR/U6. The Bel-7402 and HepG2 cells were stably transfected with the TNFAIP8-shRNA plasmids using Attractene following the manufacturer's instructions. The empty plasmid was used as a negative control. Selection was accomplished with G418 (Sigma) at a concentration of 0.4 mg/mL.

### MTT, colony formation assay, soft agar colony formation assay and Matrigel invasion assay

For MTT assay cells were plated in 96-well plates about 2000 cells per well. 24 hours after transfection. For quantification of cell viability, 20 μl of 5 mg/ml MTT (Thiazolyl blue) solution was added to each well and incubated for 4 hours at 37°C. then the medium was removed from each well, and the remaining MTT formazan was dissolved in 150 μl DMSO. Specimen was detected using a plate reader at the wavelength of 490 nm.

For colony formation assay, cells were seeded into 6-cm cell culture dishes (1000 per dish) and incubated for w weeks. Plates were washed with PBS and stained with Giemsa. The number of colonies with more than 50 cells was counted.

For soft agar anchorage independent colony growth assay, about 2000 cells per well were seeded in medium containing 0.4% agarose on top of bottom agar containing 1% low-melting agar in regular medium. After 6-8 weeks, colonies were stained with Giemsa and counted.

Matrigel invasion assay was performed using a 24-well Transwell chamber with a pore size of 8 μm (Costar), and the inserts were coated with 20 μl Matrigel (1:3 dilution, BD Bioscience). 48 hours after transfection, cells were trypsinized, transferred to the upper Matrigel chamber in 100 μl serum-free medium containing 4×10^5^ cells, and incubated for 16 hours. Medium supplemented with 15% FBS was added to the lower chamber as attractant. Cells on the upper membrane surface were removed with a cotton tip and the cells that passed through the filter were fixed in 4% paraformaldehyde and stained with hematoxylin. The experiments were performed in triplicate.

### RNA extraction and real-time RT-PCR

Total RNA was isolated from cultured cells using the RNAiso Plus reagent (TaKaRa, Dalian, China). Reverse transcription of 1 μg of RNA was performed using the PrimeScript RT Mastermix (TaKaRa, Dalian, China) according to the manufacturer's instructions. Quantitative real-time PCR was done using SYBR mastermix (TaKaRa, Dalian, China) on the 7900HT fast Real-time PCR system (Applied Biosystems). β-actin was used as the reference gene. The relative levels of gene expression were represented as ΔCt=Ct gene –Ct reference, and the fold change of gene expression was calculated by the 2^-ΔΔCt^ Method. Experiments were repeated in triplicate. The primer sequences are listed in Table 4.

**Table 4:** Primer sequences

Genes	Sequence 5’ to 3’
TNFAIP8 forward	GCCGTTCAGGCACAAAAGA
TNFAIP8 reverse	GCACCTCACTACTTGTGTCGTCTATT
Cyclin D1 forward	TGGAGGTCTGCGAGGAACA
Cyclin D1 reverse	TTCATCTTAGAGGCCACGAACAT
Cyclin E forward	AGCCAGCCTTGGGACAATAAT
Cyclin E reverse	GAGCCTCTGGATGGTGCAAT
p27 forward	CTGCAACCGACGATTCTTCTACT
p27 reverse	CTTCTGAGGCCAGGCTTCTT
CTGF forward	GTTACCAATGACAACGCCTCCT
CTGF reverse	TGCACTTTTTGCCCTTCTTAATGT
Actin forward	ATAGCACAGCCTGGATAGCAACGTAC
Actin reverse	CACCTTCTACAATGAGCTGCGTGTG

### Wound healing assay

After 24 h of growth, cells were seeded into 6-well plates at a density of about 70-90% confluence as a monolayer. The monolayer was gently scratched using a 1 ml pipette tip. Detached cells were washed away with PBS. Then the plates incubated in medium. Photos of the stained monolayer were taken using microscope. The gap distance was quantitatively evaluated using ImageJ software.

### Immunofluorescence and confocal microscopy

Cells on Lab-Tek Chamber Slides were fixed with 4% paraformaldehyde in PBS, permeabilized using 0.1% Triton X-100, blocked with serum, and then incubated with primary antibodies and AlexaFluro 488 and 555-conjugated secondary antibodies (Molecular Probes, USA). Photos was taken using Olympus FV1000 confocal microscope (Olympus, Japan).

### Immunohistochemistry

Tumor specimens were fixed with 10% neutral formaldehyde, embedded in paraffin, and sectioned 4-μm-thick. Immunostaining was performed using the 2 step elivision plus kit (MaiXin, Fuzhou, China). Tissue sections were incubated with TNFAIP8 rabbit polyclonal antibody (Abcam, 1:500 dilution), p-LATS1 polyclonal antibody (Cell signaling, 1:100 dilution) and YAP monoclonal antibody (Cell signaling, 1:100 dilution). All tumor slides were examined randomly by two independent investigators. Five views were examined per slide, and 100 cells were observed per view at 400×magnification. The intensity of TNFAIP8 cytoplasmic staining was scored as follows: 0, negative; 1, weak; 2, moderate; 3, strong. The percentage of stained tumor cells was scored as 0, 0%; 1, 1-25%; 2, 26-50%; 3, 51-75% and 4, 76-100%. The scores of each tumor sample were multiplied to give a final score of 0-12, and the tumor samples with a final score ≥6 were regarded as TNFAIP8 high expression.

### Western blot analysis and Immunoprecipitation

Total protein were extracted in lysis buffer and quantified using the Bradford method, separated by SDS–PAGE and then transferred to polyvinylidene fluoride (PVDF) membrane (Millipore, Billerica, MA, USA). The membranes were incubated overnight at 4°C with appropriate primary antibodies. TNFAIP8 rabbit polyclonal antibodies were purchased from Abcam (Cambridge, MA, USA). Antibodies against cyclin D1, cyclin E, p27, CDK4, CDK6, CTGF, p-YAP, YAP, p-LATS1, LATS1, p-MST1/2 and GAPDH were obtained from Cell Signaling Technology (Beverly, MA, USA). After incubation with peroxidase-coupled anti-mouse or anti-rabbit IgG (Cell Signaling Technology) at 37°C for 2 hours. Proteins were visualized using ECL (Pierce, Rockford, IL USA) Images were captured using DNR Bio-Imaging Systems (Jerusalem, Israel).

For immunoprecipitation, Magnetic Beads (Bio-Rad SureBeads) were incubated with antibodies and unbound antibodies were washed away. Then beads-antibody complex was incubated with target protein. The beads were magnetized using SureBeads magnetic rack and supernatant was discarded. Then elution buffer was used to collect purified target protein for western blot analysis.

### Flow cytometry for cell cycle analysis

Cells were seeded into 6 cm tissue culture dishes. 48 hours after transfection, cells were harvested, fixed in 1% paraformaldehyde, washed with PBS and stained with 5 mg/ml propidium iodide in PBS supplemented with RNase A (Roche, Indianapolis, IN) for 30 minutes at room temperature. Cells in each individual phase of the cell cycle were determined based on their DNA ploidy profile.

### Luciferase reporter assay

To assess change of Hippo signaling and YAP transcription activity, we performed luciferase assay according to the manufacturer's protocol (Promega, USA). Cells were transfected with luciferase reporter plasmid 8xGTIIC-luciferase with TNFAIP8 plamid or siRNA. 48 hours after transfection, the cells were analyzed for luciferase activities.

### *In vivo* xenograft tumor models

BALB/c athymic nude mice (4 weeks old) were purchased from Shanghai Slac Laboratory Animals Ltd. (Shanghai, China) and housed in the Laboratory Animal Center of China Medical University (Shenyang, China). All animal experiments and procedures conformed to the institutional animal care guidelines. A xenograft model of human lung cancer was established by subcutaneous right armpit injections of stable cell lines (5*10^6^). Tumor size was measured every week. Following 6 weeks' growth, animals were sacrificed and xenograft tumors were removed.

### Statistical analysis

SPSS version 16 for Windows was used for all analyses. The χ^2^-test was used to examine possible correlations between TNFAIP8 expression and clinicopathologic factors. The Kaplan-Meier method was used to estimate the probability of patient survival, and differences in the survival of subgroups of patients were compared by using Mantel's log-rank test. The Cox regression model was used for multivariate analysis. Differences between transfection/control groups were compared using Student's t-test. p<0.05 was considered as statistically significant.

## SUPPLEMENTARY MATERIALS FIGURES AND TABLES


