# Supplementary material for: TNFAIP8 interacts with LATS1 and promotes aggressiveness through regulation of Hippo pathway in hepatocellular carcinoma
Source: Oncotarget. 2017 Feb 1;8(9):15689–703. doi: 10.18632/oncotarget.14938 (PMC5362516; doi:10.18632/oncotarget.14938)
Supplement: Supplementary file 1 [file oncotarget-08-15689-s001.pdf]

TNFAIP8 interacts with LATS1 and promotes aggressiveness through regulation of Hippo pathway in hepatocellular carcinoma

SUPPLEMENTARY FIGURES

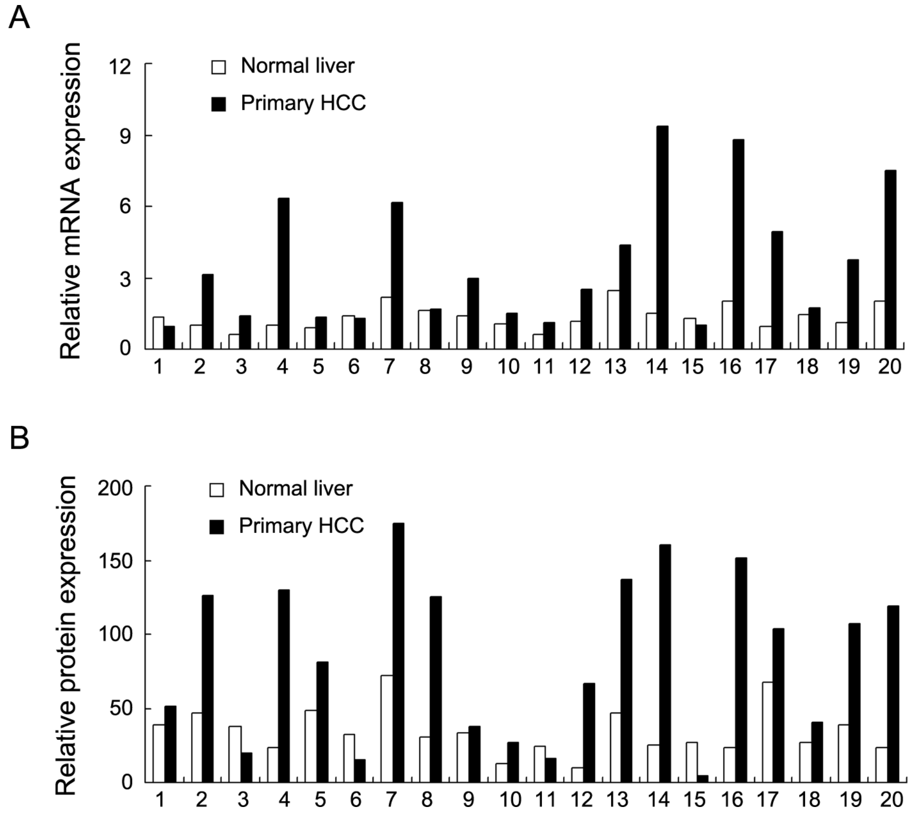

Supplementary Figure 1: Relative protein and mRNA levels in 20 paired fresh tissues. A. Individual mRNA expression in 20 cases of fresh HCC tissues and adjacent normal tissues. **B.** Protein bands were quantified and TNFAIP8 expression in each case was shown.

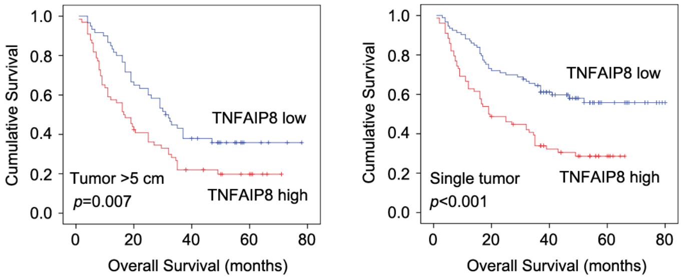

Supplementary Figure 2: Survival analysis in sub-cohort. TNFAIP8 associated with poor overall survival in HCC subgroups with single tumor and tumor size >5cm.

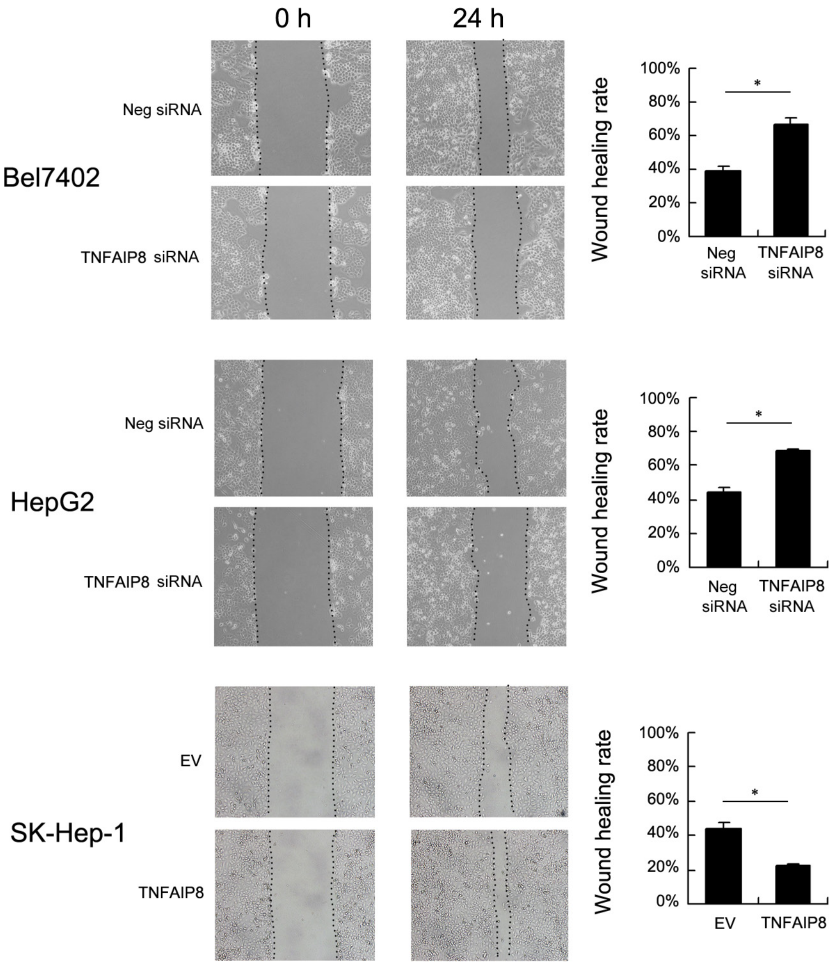

Supplementary Figure 3: Cell migration assay. Scratch test showed that TNFAIP8 siRNA attenuated cell migration in both of Bel7402 and HepG2 cells (gap distance ratio: Bel7402, control vs TNFAIP8 siRNA: 38.6%±3.3 versus 66.8%±3.9, p<0.05; HepG2, control vs TNFAIP8 siRNA: 44.3%±2.7 versus 68.7%±1.1, p<0.05). TNFAIP8 overexpression accelerated cell migration(EV vs TNFAIP8 plasmid: 43.9%±0.5 versus 22.2%±0. 2, p<0.05).

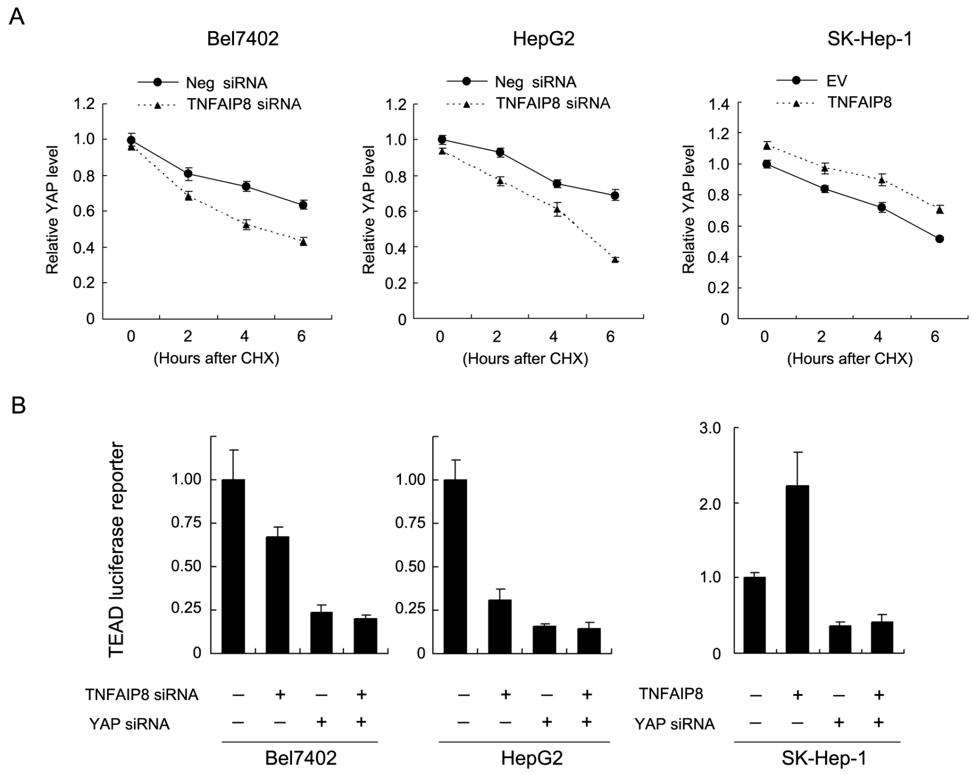

Supplementary Figure 4: TNFAIP8 regulates YAP stability. A. Protein quantification of YAP degradation treated with CHX. **B.** YAP depletion blocked the effect of TNFAIP8 siRNA/ plasmid on TEAD luciferase actibity.
